# Identifying rare diseases using electronic medical records: the example of allergic bronchopulmonary aspergillosis

**DOI:** 10.1002/pds.4204

**Published:** 2017-03-31

**Authors:** Andrew Maguire, Michelle E. Johnson, David W. Denning, Germano L.C. Ferreira, Adrian Cassidy

**Affiliations:** ^1^ OXON Epidemiology Ltd London UK; ^2^ Department of Biomedical Sciences University of Alcala de Henares Madrid Spain; ^3^ National Aspergillosis Centre University Hospital of South Manchester, The University of Manchester, Manchester Academic Health Science Centre Manchester UK; ^4^ Vaccine Discovery and Development GSK Vaccines Wavre Belgium

**Keywords:** allergic bronchopulmonary aspergillosis, electronic medical records, burden, case definition, rare disease, CPRD, pharmacoepidemiology

## Abstract

**Purpose:**

The purpose of the study is to evaluate whether primary care electronic medical records (EMRs) from patients with severe asthma can be used to identify allergic bronchopulmonary aspergillosis (ABPA) cases.

**Methods:**

This cross‐sectional feasibility study was conducted in adults with active and severe asthma registered with the Clinical Practice Research Datalink. A set of keywords flagged terms potentially indicative of ABPA in free‐text comments of patients' EMRs to produce a grid on the basis of keywords' hit or miss. The grid was examined for occurrence and concurrence of keywords to discern patterns of concurrence potentially indicative of an underlying diagnosis of ABPA.

**Results:**

The analyses included 3 653 169 free‐text items from 21 054 patients. In total, 52 patients (0.25%) had at least one mention of ‘ABPA’ in their medical record; 67% of these patients also had a mention of ‘aspergillus/aspergillosis’, 54% of ‘bronchiectasis’, 42% of ‘itraconazole’ and 62% of ‘IgE’. The term ‘aspergillus/aspergillosis’ occurred with a proportion of 1.84% (*N* = 387); 9% of these patients also had a mention of ‘ABPA’, and the remaining 91% were potential additional cases of ABPA. From the observed concurrence of keywords, we were able to devise a potential algorithm to identify cases with varying degrees of specificity.

**Conclusions:**

This study suggests that analysis of free text within asthmatic patients' EMRs may be used to identify potential cases of ABPA. This could be an efficient approach to identify rare conditions and to quantify their potential burden. © 2017 The Authors. Pharmacoepidemiology & Drug Safety Published by John Wiley & Sons Ltd.

## Introduction

Allergic bronchopulmonary aspergillosis (ABPA) is characterised by a hypersensitivity reaction to *Aspergillus fumigatus* 1, 2 and occurs almost exclusively in patients with asthma or cystic fibrosis.^1^, ^3^, ^4^ Recent estimates suggest that approximately 5.4 million patients have been diagnosed with asthma in the UK, of which around 4.3 million are adults.^5^, ^6^ The highest prevalence of ABPA is seen in severe asthma patients, and this condition can create further complications such as poorly controlled asthma, airway obstruction with mucous plugging mimicking pneumonia, bronchiectasis and chronic pulmonary aspergillosis.^3^, ^7^ Although disease exacerbations can be prevented by early treatment, ABPA is often undetected and hence untreated in asthma patients. The diagnosis of this condition is currently based on clinical, radiological and immunological (immunoglobulin E [IgE] levels) findings, but there is no consensus on the criteria and cut‐off values of IgE levels required.^1^

To our knowledge, no population‐based study has evaluated the epidemiology and burden of ABPA. However, in a previous study using data from five referral cohort studies (China, Ireland, New Zealand, Saudi Arabia and South Africa), the prevalence of ABPA in adults with asthma was estimated at 2.5% (range: 0.72–3.50%).^7^, ^8^, ^9^, ^10^, ^11^, ^12^ In another case series study in Iran, the prevalence of ABPA among asthmatic patients was estimated at 4.1%.^13^ In these studies, the prevalence could be overestimated because estimates were based on case series seen in secondary care clinics, where patients have more severe asthma, probably in part caused by ABPA infection. On the other hand, rates could be underestimated as the condition is chronic and most studies simply examine referrals over a limited time period.

Given that ABPA is not routinely coded by physicians, electronic medical records (EMRs) could feasibly be used to define a set of symptoms, test results and other relevant information to identify patients with asthma likely to be undergoing an ABPA episode. The identification of ABPA diagnoses using EMRs would enable the burden of ABPA to be estimated, providing key information on the epidemiology of this rare disease. In this study, we evaluated whether it is possible to use EMRs to identify patients with a rare condition, such as ABPA, in the absence of a gold standard for the diagnosis.

## Methods

### Study design

This was a cross‐sectional feasibility study to evaluate whether free‐text information recorded within a primary care EMR can be used to identify potential cases of ABPA among patients with asthma.

Whilst a representative population of asthma patients is readily identifiable within primary care databases of the UK, the identification of ABPA is not straightforward. Although ABPA has a unique code according to the International Classification of Diseases (ICD‐10 code: B44.81),^14^ no specific Read codes for ABPA are currently available. Therefore, a broader algorithmic approach was required to identify cases in the Clinical Practice Research Datalink (CPRD) in the UK.

The CPRD has been granted Multiple Research Ethics Committee approval (05/MRE04/87) to undertake purely observational studies, with external data linkages including Hospital Episode Statistics and Office for National Statistics mortality data. The work of the CPRD is also covered by the National Information Governance Board—Ethics and Confidentiality Committee approval (ECC 5‐05 (a) 2012). This study was endorsed by the Independent Scientific Advisory Committee (ISAC) for Medicines and Healthcare Products Regulatory Agency database research (ISAC protocol number: 15_104R; protocol available upon request). Although this study was based in part on data from the CPRD obtained under license from the UK Medicines and Healthcare Products Regulatory Agency, the interpretation and conclusions contained in this article are the sole responsibility of the authors.

### Study population

The study population was defined as adults with active and severe asthma at the index date (1 July 2013), who were registered with one of the general practitioner (GP) practices contributing to the CPRD in the UK. Patients were included if they had a registration date with the CPRD prior to 1 July 2011 and were still active on 1 July 2013 (i.e. had at least 2 years of medical history), were up to standard for CPRD research and had ≥1 prescription for an asthma medication any time between 1 July 2012 and 1 July 2013.

To minimise the risk of misclassification the analysis did not include patients with other conditions potentially associated with ABPA, such as cystic fibrosis, or patients with conditions that may be misdiagnosed as ABPA and appear in the differential diagnosis, such as sarcoidosis. Patients were therefore excluded if they were less than 18 years of age at index date, had a record of chronic obstructive pulmonary disease, cystic fibrosis, human immunodeficiency virus, organ transplant or sarcoidosis anytime in their medical records prior to index date or if they did not have active or severe asthma at index date.

Active asthma was defined as patients with ≥2 prescriptions for short‐acting beta‐agonist, inhaled corticosteroids or long‐acting beta‐agonist during the 12‐month period prior to index date. These drugs were selected because they are specific to asthma (i.e. not indicated for chronic obstructive pulmonary disease) and should have captured all active asthma cases. Owing to the higher prevalence of ABPA in more severe asthma patients, the most restrictive definition of severe asthma was used (i.e. patients who needed therapy beyond the short‐acting beta‐agonist + inhaled corticosteroids + long‐acting beta‐agonist regimen or with a record of asthma exacerbation during the last 3 months).^15^, ^16^

### Identification of ABPA

The CPRD is a UK primary care database. At the time of data extraction, it was collecting data from 700 practices in the UK, covering approximately 5.7 million active patients. The CPRD contains data from pseudonymised patients and includes demographics and all care events that GPs have chosen to record as part of their usual medical practice using Read diagnosis codes complemented by free‐text notes.^17^ Free‐text information is used by GPs to record notes regarding a patient consultation and any other additional information they feel to be of relevance to the patient. In addition, free text can include scanned letters from specialists and therefore may contain additional information related to ABPA received from hospitalisation or outpatient specialist care.

To develop an ABPA case‐finding algorithm, a list of clinically relevant terms and keywords in ABPA diagnosis was developed and reviewed by a clinical expert (Supplement 1). This list was based on pre‐specified keywords including diagnostic terms (ABPA, aspergillosis, invasive [used to rule out invasive aspergillosis] and fungal [mentions of general fungal infection]), symptoms or manifestations (eosinophilia, bronchiectasis and exacerbations), tests (IgE, immunoglobulin and skin prick test) and treatment (itraconazole, voriconazole and other antifungals). A final set of 11 keywords was created by checking, for example, if some words in Supplement 1 could be collapsed. Because it is unlikely that the occurrence of ABPA represented letters as part of another word, the occurrence of ABPA without considering its position in the free‐text was used; this is the ‘abpa_all’ variable described in Supplement 1. In contrast, IgE was considered if it occurred immediately after a space or if it occurred at the beginning of the free‐text string, because the letters ‘ige’ are common in other words. No items of free‐text that included the keyword ‘invasive’ were found, and hence, this word was not considered.

The list of keyword text strings potentially indicative of ABPA diagnosis was provided to the CPRD research team to run an automated query on the free‐text comments available in the patients' records. Free‐text information from the 5 years prior to the index date (1 July 2013) was searched and flagged for the keyword markers, following the instructions given in Supplement 1. The CPRD research team performed the free‐text search, produced a grid on the basis of a keyword hit or miss among the free‐text comments and provided the length of each free‐text comment. The distribution of the occurrence and concurrence of the keywords was evaluated by a clinical expert to assess the likelihood that patients could be suffering from ABPA, albeit undiagnosed. The study team had no access to the free text.

### Statistical analyses

In previous reports, ABPA prevalence in asthma patients may have been overestimated because of selection bias. However, even if the underlying prevalence was 10‐fold lower than the reported average prevalence of 2.5%, the probability of observing at least one ABPA case was 95% with a sample size of 1200 patients. Because the estimated sample size was likely to exceed 20 000 patients with severe asthma, the probability to have prevalent cases of ABPA in the CPRD primary care population was high.

In order to select the items of free‐text for subsequent review, the occurrence and concurrence of the keywords were examined. Free‐text strings with no keywords were not considered further. Among the remaining free‐text strings, the occurrence of each of the keywords was summarised, and the concurrence of the keywords was described. For this study, the analysis was descriptive and hypothesis generating. The concurrence and occurrence of the keywords in free text were summarised using a contingency table.

The programming was conducted, and the data were analysed using Statistical Analysis System (sas) software version 9.3 (SAS Institute Inc., Cary, NC, USA).

## Results

### Demographics of the asthma population for free‐text review

**Figure 1:**
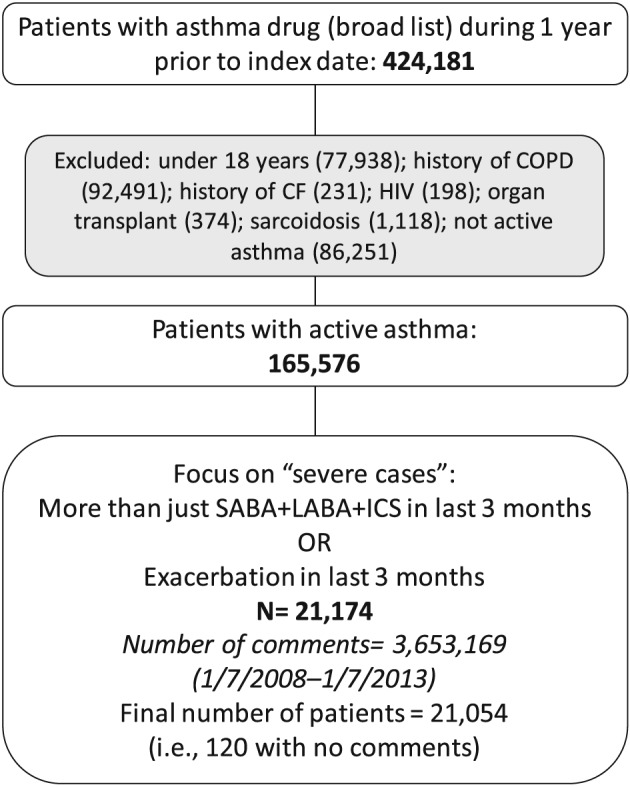
Flow chart of patient selection process. CF, cystic fibrosis; COPD, chronic obstructive pulmonary disease; HIV, human immunodeficiency virus; ICS, inhaled corticosteroids; LABA, long‐acting beta‐agonist; SABA, short‐acting beta‐agonist

The total number of asthma patients identified was 424 181 (Figure 1). The study population was restricted to 165 576 adults with active asthma, who were not excluded on the basis of the confounding conditions. From the 165 576 patients, 21 174 patients with evidence of severe asthma were included. A total of 3 653 169 items of free‐text from 21 054 patients were included in the analyses.

### Description of the occurrence and concurrence of keywords

**Figure 2:**
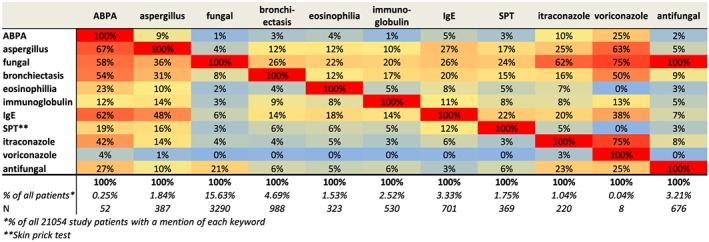
Occurrence and concurrence of keywords over the previous 5 years for patients with current severe asthma. ABPA, allergic bronchopulmonary aspergillosis; IgE, immunoglobulin E. [Colour figure can be viewed at wileyonlinelibrary.com]

A contingency table was developed using 11 keywords, and rates of occurrence were calculated as percentages from the 21 054 severe asthma patients (Figure 2). The results represent the univariate occurrence and the two‐way concurrence of each keyword.

In total, 52 patients had at least one mention of ‘ABPA’ in their medical record, suggesting a low use of this term (0.25%) (Figure 2). Among patients with a mention of ‘ABPA’, 67% also had a mention of ‘aspergillus/aspergillosis’, 54% of ‘bronchiectasis’, 42% of ‘itraconazole’ and 62% of ‘IgE’. The term ‘aspergillus/aspergillosis’ occurred with a proportion of 1.84% (*N* = 387); 9% of these patients also had a mention of ‘ABPA’, and the remaining 91% may include additional cases of ABPA, as a potential ABPA diagnosis was supported by a concurrence of 31% with bronchiectasis and 48% with IgE.

The proportion of patients with at least one mention of ‘fungal’ was 15.63% (*N* = 3290). The concurrence of 21% with ‘antifungal’ suggested that ≥79% of the mentions of fungal referred to fungal infection rather than antifungal treatment.

The prevalence of the terms ‘bronchiectasis’ and ‘eosinophilia’ was 4.69% (*N* = 988) and 1.53% (*N* = 323), respectively. The concurrence of ‘bronchiectasis’ with ‘fungal’, ‘IgE’ and ‘aspergillus/aspergillosis’ was 26%, 14% and 12%, respectively. The concurrence of ‘eosinophilia’ with ‘fungal’, ‘IgE’ and ‘aspergillus/aspergillosis’ was 22%, 18% and 12%, respectively.

Overall, ‘IgE’ was mentioned in 3.33%, ‘immunoglobulin’ in 2.52% and ‘skin prick tests’ in 1.75% of patients. These terms are of limited value in the absence of test results, but they could reflect suspicion of a fungal infection. Around 1% of patients had a mention of ‘itraconazole’; 25% of these patients had a specific mention of ‘aspergillus/aspergillosis’.

### Proposal for on the development of an ABPA case‐finding algorithm in the free‐text of electronic medical records

Building blocks of an algorithm were identified on the basis of the natural order of the occurrence and concurrence of keywords in terms of their specificity related to the diagnosis of ABPA. Increasing values of ABPA flags (1 to 4) denote decreasing specificity of a potential ABPA diagnosis.

A flag value of 1, meaning likely ABPA case, was assigned to patients with a mention of ‘ABPA’. A flag value of 2 was assigned to patients with no mention of ‘ABPA’, but with a mention of ‘aspergillus/aspergillosis’, and a mention of ‘bronchiectasis’ or ‘eosinophilia’. A flag value of 3 was assigned to the other patients with a mention of ‘aspergillus/aspergillosis’. Finally, patients with a mention of ‘fungal’ and a mention of ‘bronchiectasis or eosinophilia’ received a flag value of 4. It would also be advisable to consider the chronological concurrence of terms. For example, if a mention of ‘fungal’ was close in time to a mention of ‘bronchiectasis’, an ABPA diagnosis would be more likely than if the terms occurred several years apart.

## Discussion

This study suggests that free‐text information in EMRs may be used to identify potential ABPA cases in patients with severe asthma. An algorithm including the observed combinations of the occurrence of keywords related to ABPA allowed the identification of potential cases of ABPA. Identification of ABPA patients would then enable studies that use the richness of the primary care record and also allow for patient and physician questionnaires to be administered. However, subsequent validation is required, possibly via GP questionnaires, clinical review of primary care notes and linkage of primary and secondary care records to ascertain its positive predictive value.

In the study population of patients with current and severe asthma, the term ‘ABPA’ was not frequently mentioned in the free‐text (0.25%). This proportion is much lower than the prevalence of ABPA observed in a series of case studies (range: 0.72–4.10%).^7^, ^8^, ^9^, ^10^, ^11^, ^12^ However, when mentioned, the term ‘ABPA’ coincided with other terms also indicative of ABPA, which is suggestive that the mention of ABPA may have represented a true diagnosis. The low frequency of use of ‘ABPA’ in the population at highest risk also means that it is necessary to rely on other related terms to identify potential cases of this infection.

Besides the term ‘ABPA’, other words potentially indicative of ABPA were identified, such as ‘aspergillus’, ‘aspergillosis’, ‘bronchiectasis’ or ‘eosinophilia’. The low concurrence of ABPA‐related terms with the word ‘fungal’ indicated that ‘fungal’ is likely to be too unspecific and not sufficient to identify ABPA, even among patients with severe asthma. Some of the cases with a mention of ‘fungal’ may be identifiable through other concurrent keywords, whilst others would need to be identified through an evaluation of the depersonalised content of the free‐text comments. In particular, the concurrence of ‘fungal’ with ‘bronchiectasis’ or ‘eosinophilia’ may be sufficiently specific to infer ABPA. Further research should test the validity of the main keywords identified in this study. Specifically, it would be important to understand the real context of their occurrence, which may require access to the patient's medical record.

The most important limitation of this study was the lack of recording of key diagnostic details. For example, it was not possible to evaluate the proportion of patients who had IgE test results, because results of these tests are not always transmitted to GPs when they are performed in hospital or are not recorded by GPs when they are not remarkable. The unstructured data used in this study relied on written information provided by GPs in free‐text fields that could lead to inference of an ABPA diagnosis and on information scanned from specialist letters received by GPs. Moreover, the algorithm that was developed did not allow the identification of all patients with ABPA, particularly those excluded because their asthma was not active or severe at index date. A further limitation was that the concurrence of keywords may not occur in the same string of free text, and there could be a gap of up to 5 years between the occurrences of the words. The choice of time frame for free‐text evaluation was a balance between reducing the number of free‐text comments and obtaining enough evidence from a period that would reflect the potential impact of ABPA on the severity of the patients' condition. Lastly, given cultural differences and also due to the availability of free‐text, the conclusion of this study should only be considered within the context of the UK health system, although ABPA is a well‐accepted term worldwide in English.

Besides these limitations, this study also has several strengths, such as the large sample size, which is important for rare conditions such as ABPA. Moreover, the selected study population was in many respects representative of the UK population of asthma patients, and data collected in routine clinical practice allowed the generation of real‐world evidence. In the UK, GPs have a role of gatekeeping that maximises the completeness of the data available in the medical records, and the condition of asthma is part of the Quality and Outcomes Framework, where high quality data are audited.

The method used in this study to analyse free‐text comments relied on the automated screening of the free‐text notes performed by the database custodian CPRD research team. Free‐text medical notes were not transferred to the research team to minimise any potential breach of patient confidentiality. Such a method benefits healthcare research whilst complying with the latest data protection regulations and provisions of CPRD. Although there is uncertainty surrounding the future availability of free‐text information in CPRD and other primary care data, this study has demonstrated that free‐text comments can identify rare conditions such as ABPA and that the scope of healthcare research would be severely restricted without access to such information. This is potentially an efficient approach to identify such a rare condition and to quantify its potential burden. Other rare diseases could benefit from a similar approach whereby the frequency of keywords and their concurrence will lead to an initial decision as to whether such databases and their free‐text hold relevant data that could enable detection of rare diseases.

## Conflict of Interest

The employer of Andrew Maguire and Michelle E Johnson (OXON Epidemiology Ltd) was funded by the GSK group of companies to undertake the study design and analyses of this project. At the time of the study, Germano LC Ferreira and Adrian Cassidy were employees of the GSK group of companies. David W Denning holds founder shares in F2G Ltd and Novocyt and has current grant support from the National Institute of Health Research, the Medical Research Council, the Global Action Fund for Fungal Infections and the Fungal Infection Trust. He acts or has recently acted as a consultant to Astellas, Sigma Tau, Basilea, Scynexis, Cidara and Pulmocide. In the last 3 years, he has been paid for talks on behalf of Astellas, Dynamiker, Gilead, Merck and Pfizer. He is also a member of the Infectious Disease Society of America Aspergillosis Guidelines and European Society for Clinical Microbiology and Infectious Diseases Aspergillosis Guidelines groups. Adrian Cassidy holds shares in the GSK group of companies.

**Key Points**

- Analyses of free‐text comments in electronic medical records may be used to identify potential allergic bronchopulmonary aspergillosis (ABPA) cases in patients with severe and active asthma.
- The identification of ABPA diagnoses using electronic health data would enable the burden of ABPA to be estimated nationally, providing key information on the diagnosis and therapy gap for this uncommon disorder.

## Author Contributions

All authors were involved in the conception or the design of the study, participated in the collection or generation of the data, contributed to the materials/analysis tools and were involved in the analyses and interpretation of the data. A. F. C., M. E. J. and A. M. performed the study. All authors reviewed and commented on manuscript drafts and gave final approval for it to be submitted for publication.

## Supporting information


**Supplement 1**. Keywords and logic algorithm to flag relevant free‐text from the CPRD
