# Supplementary material for: Identifying rare diseases using electronic medical records: the example of allergic bronchopulmonary aspergillosis
Source: Pharmacoepidemiol Drug Saf. 2017 Mar 31;26(7):785–91. doi: 10.1002/pds.4204 (PMC5573984; doi:10.1002/pds.4204)
Supplement: Supplementary file 1 — Supplement 1. Keywords and logic algorithm to flag relevant free‐text from the CPRD [file PDS-26-785-s001.docx]

**Supplement 1. Keywords and logic algorithm to flag relevant free-text from the CPRD**

1. Output the length (number of words)
   - output variable name: “Length”: numerical value of the number of words in the string
2. Search for exact string “*abpa*”:
   - output variable name: “ABPA_all”: if string present then assign 1=yes; else 0=no
3. Search for exact string “* abpa*” [note space]:
   - output variable name: “ABPA_with_space”: if string present then assign 1=yes; else 0=no
4. Search for exact string “abpa*” [used to capture when appears as first word in free-text]:
   - output variable name: “ABPA_1st_word”: if string present then assign 1=yes; else 0=no
5. Search for exact string “*aspergill*” [hope to capture either aspergillus or aspergillosis]:
   - output variable name: “Aspergillus”: if string present then assign 1=yes; else 0=no
6. Search for exact string “*invasive*”:
   - output variable name: “Invasive”: if string present then assign 1=yes; else 0=no
7. Search for exact string “*fung*”:
   - output variable name: “Fungal”: if string present then assign 1=yes; else 0=no
8. Search for exact string “*bronchiectasis*”:
   - output variable name: “Bronchiectasis”: if string present then assign 1=yes; else 0=no
9. Search for exact string “*eosinophilia*”:
   - output variable name: “Eosinophilia”: if string present then assign 1=yes; else 0=no
10. Search for exact string “*exacerbat*”:
    - output variable name: “Exacerbation”: if string present then assign 1=yes; else 0=no
11. Search for exact string “*immunoglo*”:
    - output variable name: “Immunoglobulin”: if string present then assign 1=yes; else 0=no
12. Search for exact string “* ige*” [note space]:
    - output variable name: “lgE”: if string present then assign 1=yes; else 0=no
13. Search for exact string “ige*” [used to capture when appears as first word in free-text]:
    - output variable name: “IgE_1st_word”: if string present then assign 1=yes; else 0=no
14. Search for exact string “* spt*” [note space] or “*skin prick*”:
    - output variable name: “SPT”: if string present then assign 1=yes; else 0=no
15. Search for exact string “*itraconazole*” or “*sporanox*”:
    - output variable name: “Itraconazole”: if string present then assign 1=yes; else 0=no
16. Search for exact string “*voriconazole*” or “*vfend*”:
    - output variable name: “Voriconazole”: if string present then assign 1=yes; else 0=no
17. Search for exact string “*antifung*” or “*antimycot*”:
    - output variable name: “Antifungal”: if string present then assign 1=yes; else 0=no
